# Improving Photophysical Properties of White Emitting Ternary Conjugated Polymer Blend Thin Film via Additions of TiO_2_ Nanoparticles

**DOI:** 10.3390/polym12092154

**Published:** 2020-09-21

**Authors:** Sameer Al-Bati, Mohammad Hafizuddin Hj. Jumali, Bandar Ali Al-Asbahi, Khatatbeh Ibtehaj, Chi Chin Yap, Saif M. H. Qaid, Hamid M. Ghaithan, W. A. Farooq

**Affiliations:** 1 Department of Applied Physics, Faculty of Science and Technology, Universiti Kebangsaan Malaysia, UKM Bangi 43600, Selangor, Malaysia; p90632@siswa.ukm.edu.my (K.I.); ccyap@ukm.edu.my (C.C.Y.); 2 Department of Physics and Astronomy, College of Science, King Saud University, Riyadh 11451, Saudi Arabia; sqaid@ksu.edu.sa (S.M.H.Q.); 436107632@student.ksu.edu.sa (H.M.G.); awazirzada@ksu.edu.sa (W.A.F.); 3 Department of Physics, Faculty of Science, Sana’a University, Sana’a P.O. Box 12544, Yemen; 4 Department of Physics, Faculty of Science, Ibb University, Ibb P.O. Box 70270, Yemen

**Keywords:** Förster Resonance Energy Transfer, nanocomposites, exciplex inhibition, charge trapping effect

## Abstract

The effect of TiO_2_ nanoparticles on the photophysical properties of ternary conjugated polymer (CP) blends of poly(9,9-dioctylfluorene-2,7-diyl) (PFO), poly 9,9-dioctylfluorene-alt-benzothiadiazole (F8BT) and poly(2-methoxy-5(2-ethylhexyl)-1,4 -phenylenevinylene (MEH-PPV) thin films was investigated. This ternary blend used a fixed amount of PFO as the donor with MEH-PPV and F8BT in various ratios as the acceptors. The solution-blending method and the spin-coating technique were used to prepare the blends and the thin films, respectively. Through efficient Förster Resonance Energy Transfer (FRET), the desired white emission was achieved with PFO/0.3 wt.% F8BT/0.5 wt.% MEH-PPV ternary blend thin film. Additions of nanoparticles up to 10 wt.% dramatically intensified the white emission which then dimmed at higher contents due to agglomerations. The current density–voltage characteristics of the nanocomposite thin films exhibited dependency on the content and distributions of the nanoparticles. Finally, a possible underlying mechanism for the intensification of emission is proposed.

## 1. Introduction

Advancements in material engineering and fabrication technologies have enabled giant electronics companies to offer high-quality organic light emitting diodes (OLEDs) screens for various applications such as in televisions and phones. From the material aspect, success in the production of high-quality displays is a reward of relentless effort in the investigation of conjugated polymers (CPs). This is because, CPs have several advantages such as better emission tunability, low cost and simple fabrication [1,2].

In practice, light emissions from OLEDs can be tuned either by blending compatible CPs or controlling the energy transfer between the CPs or a combination of both [3,4,5]. For example, a full-color emitter for white OLEDs (WOLEDs) can be achieved by blending different CPs that emit lights covering the entire visible spectrum. Currently, most WOLEDs are prepared by blending two CPs that emit blue light and red or yellow light. However, prolonged stability, luminance and efficiency issues with binary WOLEDs inspire scientists and researchers to continue their quest for different CPs combinations with better properties.

One well-studied binary blend WOLED is the combination of poly(9,9-dioctylfluorene-2,7-diyl) (PFO) and poly(2-methoxy-5(2-ethylhexyl)-1,4-phenylenevinylene (MEH-PPV) that produces blue and red emissions, respectively. In this blend, PFO with larger energy band gap acts as a donor while MEH-PPV is the acceptor. Over the years, numerous efforts have been made to improve the luminance of this binary blend WOLED by either improving the charge carrier transportation through modification of electrodes [6,7], or through blending with metal oxide nanoparticles [8,9].

Despite these efforts, the efficiency of this binary blend WOLED remains low due to the lack of green emission. A recent study on the effect of the F7GA as a green dye on the photoluminescence characteristics of the PFO/MEH-PPV blend successfully improved energy transfer efficiency between these CPs [10]. Unfortunately, this led to fast emission quenching in PFO. This problem possibly stems from the presence of two acceptors (F7GA and MEH-PPV) in this ternary blend.

Another green emitter that can be added into the original binary blend is poly 9,9-dioctylfluorene-alt-benzothiadiazole (F8BT) which is a derived copolymer of PFO. Earlier works on the absorption [11] and emission spectra [12] of F8BT reported huge spectral overlap with emission spectrum of PFO and absorption spectrum of MEH-PPV, respectively. These overlaps are indicative of promising efficient energy transfer between these CPs. The calculated Förster radii successfully proved Förster Resonance Energy Transfer (FRET) as the energy transfer mechanism in both blends [13,14]. Therefore, based on both studies, non-radiative energy transfer from PFO to F8BT and from F8BT to MEH-PPV in the PFO/F8BT/MEH-PPV ternary blend can be expected.

It is well accepted that the presence of an acceptor in any binary CPs blend will always quench the emissions from the donor [15,16,17]. Justifiably, a more dramatic quenching was reported once another acceptor is included in the initial binary blend [4,10,18]. Therefore, to achieve white emissions from PFO/F8BT/MEH-PPV ternary blend, it is of primary importance to precisely balance the delicate ratio between the CPs to avoid complete emission quenching, in particular from the PFO.

Earlier efforts to overcome this drawback in the binary blends proved that the addition of metal oxide nanoparticles, particularly TiO_2_ effectively inhibited the emission quenching. More interestingly, the presence of the nanoparticles at an appropriate amount stimulated the creation of excitons specifically in the donor and thus improved the overall emissions intensities of the blend [8,19,20]. The electron trapping effect has been proposed as the underlying explanation for the observed improvement. Thus, addition of nanoparticles may offer a feasible solution to the expected emission reduction in the PFO/F8BT/MEH-PPV ternary blend.

The current work is establishing the effects of two acceptors on the emission characteristics of the PFO/F8BT/MEH-PPV ternary blend thin films. In addition, this work also embarks on evaluating the feasibility of TiO_2_ nanoparticles as emission quenching inhibitors as well as emission enhancers for the ternary blends. Finally, the consequence of TiO_2_ nanoparticles’ presence on the surface morphology, optophysical properties and current density–voltage (J-V) characteristics of the ternary blend thin films were also investigated.

## 2. Materials and Methods

### 2.1. Materials

All materials, namely PFO, MEH-PPV, F8BT and TiO_2_ nanoparticles with a particle size of 25 nm were purchased from Sigma Aldrich, Saint Louis, MO, USA and used in this work without further purification. This work used toluene (Fluka Chemie GmbH, Buchs, Switzerland) to dissolve the CPs. Glass slides and ITO-coated glass slides with a sheet resistance of 25 Ω/sq (Merck Balzers, Balzers, Liechtenstein) were used as the substrates for the films.

### 2.2. Samples Preparation and Characterizations

All solutions were prepared using solution blending method. Stock solutions for the three CPs were prepared separately and stirred at 700 rpm for 1 h before sonication for another hour. Then MEH-PPV stock solutions were added into the PFO solution to form binary blends with different MEH-PPV wt.%. In this work, the added MEH-PPV wt.% varied between 0.1 and 0.6 wt.%. Next, 100 µL of blended solutions was deposited onto 1.5 × 2 cm^2^ glass substrates using spin coating technique at 2000 rpm for 30 s. To ensure the complete evaporation of the solvent, the thin films were left to dry at 80 °C for 1 h in an oven. Finally, all the thin film samples were subjected to absorption and emission characterizations using Lambda 950 UV-Vis-NIR spectrometer (Perkin Elmer, Waltham, MA, USA) and FLS920 photoluminescence spectrometer (Edinburgh Instruments, Livingston, UK), respectively. All photoluminescence (PL) spectra were collected using an excitation wavelength of 370 nm.

The whole process was repeated for the preparation of the ternary blend system, in which F8BT stock solutions were added into the selected PFO/MEH-PPV binary blend based on its emission profile. In this work, the added F8BT wt.% was carefully varied between 0.1 and 3.0 wt.%. Finally, different wt.% of TiO_2_ nanoparticles (ranging from 5 to 30 wt.%) were added into the selected ternary blend based on the emission profiles. Throughout the sample preparation procedures, the concentration of PFO in toluene was kept constant at 15 mg/mL.

Surface morphology and topography of selected thin films were collected using a field emission scanning electron microscope (FE-SEM) (Carl Zeiss, Supra 55VP; Cambridge, UK) and scanning probe microscope (SPM) (Park system, Nx-10; Suwon, Korea), respectively. Finally, J-V characteristics of the nanocomposite thin films were measured using Keithley 238 measurement system (Cleveland, OH, USA). For this measurement, the nanocomposite blends were spin coated onto ITO-coated glass substrates before a thin aluminum layer was thermally deposited on top of the films to act as a cathode.

## 3. Results and Discussion

### 3.1. Binary System PFO/MEH-PPV

**Figure 1:**
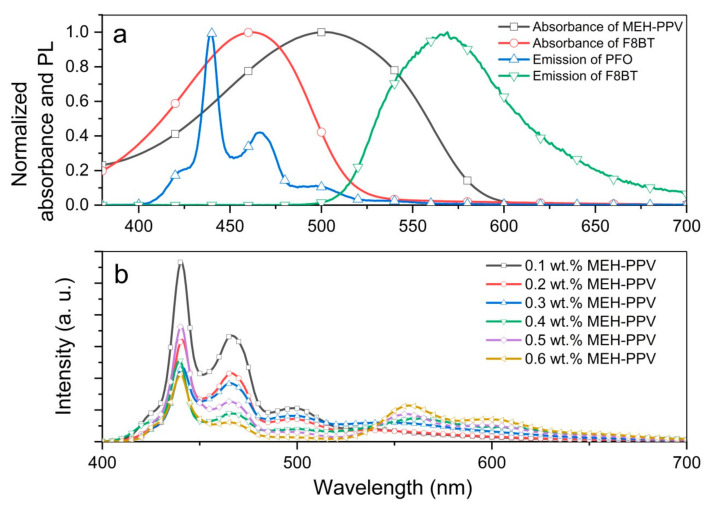
(**a**) The absorption and emission spectra of individual polymers; (**b**) emission spectra of PFO/MEH-PPV-blended thin film with various weight ratios of MEH-PPV.

The absorption and emission spectra of individual polymers used in this work are presented in Figure 1a. Clearly, there is a significant overlap between the emission spectrum of PFO and absorption spectrum of MEH-PPV, indicating the potential FRET mechanism in the blend. Actual manifestation of FRET was observed in the emission spectra of the blended binary polymer thin films (Figure 1b). It is obvious that the blue emission of PFO was successively reduced and the red emission of MEH-PPV was enhanced by the systematic addition of MEH-PPV. The current findings are in good agreement with earlier observation which proved FRET in this binary blend through Förster radius calculations [8].

### 3.2. PFO/F8BT/MEH-PPV Ternary System

**Figure 2:**
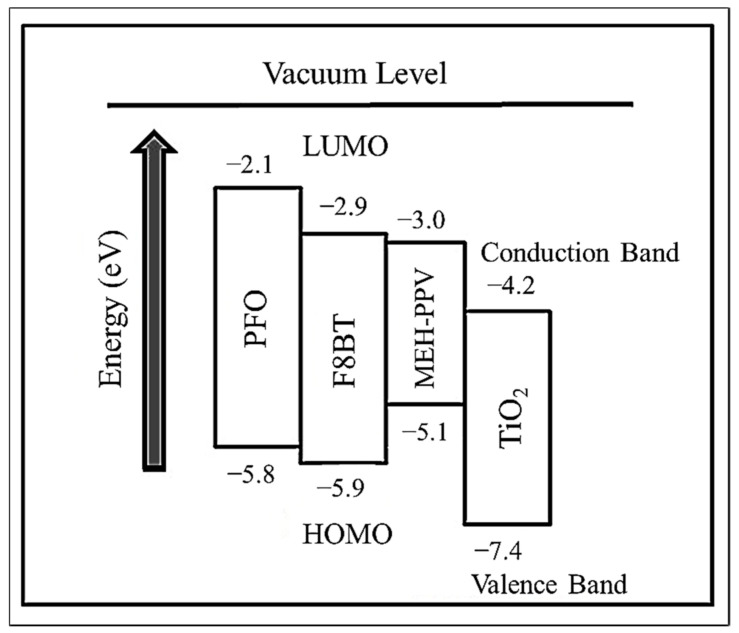
The energy level diagram of the polymers and TiO_2_.

Figure 2 illustrates the HOMO and LUMO levels of the three CPs used in this work. In addition, the valence and conduction bands of TiO_2_ are also shown [21,22,23,24]. Among the CPs, PFO has the highest LUMO of −2.1 eV while F8BT has the lowest HOMO of −5.9 eV. The former is the best hole acceptor while the latter is the best electron acceptor. The third CP namely MEH-PPV has the HOMO and LUMO of −5.1 eV and −3.0 eV, respectively. Based on the energy diagram, the selection of the CPs for the current work satisfies the two important conditions for energy transfer [11].

In a blended three polymers system (one donor and two acceptors), the fast energy transfer from the donor (usually large band-gap polymer) to the acceptors (lower band-gap polymers) rapidly quenches the donor’s emission [10,23]. To realize the desired white emission from this system, the energy transfer rate between the polymers must be carefully controlled through precise introduction of acceptors beginning from low concentration. In the PFO/F8BT/MEH-PPV ternary blend, it is empirically crucial to choose an appropriately low wt.% of MEH-PPV from the primary PFO/MEH-PPV blends before any addition of F8BT. The selected combination ratio must not cause substantial PFO emission quenching but at the same time should maintain efficient energy transfer to MEH-PPV. Therefore, the emission intensity of the PFO should be kept significantly higher than MEH-PPV to ensure sufficient energy is transferred to the second acceptor, namely F8BT. Moreover, huge spectral overlap between the emission of PFO and absorption of F8BT manifests that a large fraction of energy shall be transferred to the latter. The same strategy has been employed in a previous study [18]. Using the same criteria and referring to the emission spectra of PFO/MEH-PPV shown in Figure 1b, the MEH-PPV of 0.3 wt.% was chosen. Subsequently F8BT was added into this binary blend in small increments (starting from 0.1 wt.%) to search for the desired white emission spectrum.

#### 3.2.1. Effect of F8BT Ratio

**Figure 3:**
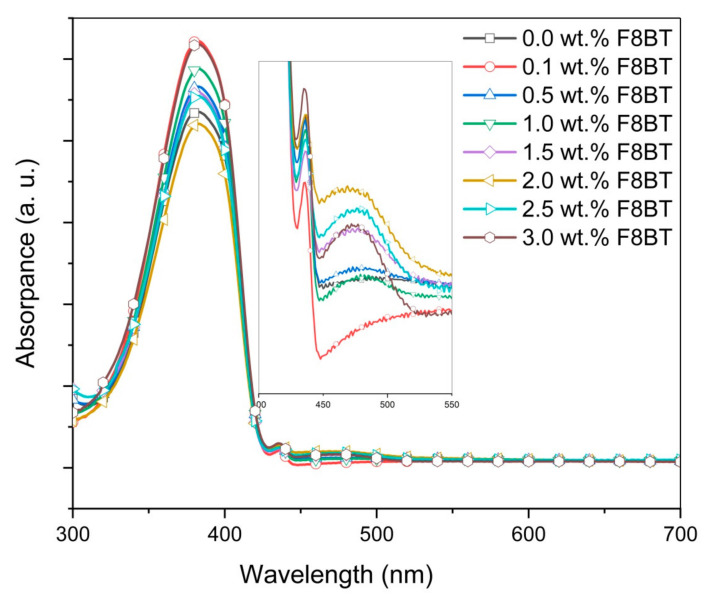
Absorbance spectra of poly(9,9-dioctylfluorene-2,7-diyl) (PFO)/poly 9,9-dioctylfluorene-alt-benzothiadiazole (F8BT)/0.3 wt.% poly(2-methoxy-5(2-ethylhexyl)-1,4 -phenylenevinylene (MEH-PPV) films with different wt.% of F8BT.

Figure 3 displays the absorption spectra of the PFO/F8BT/MEH-PPV ternary blend thin films with different concentrations of F8BT. It is obvious that the main absorption peak essentially remains unchanged with the additions of F8BT. A small peak at 435 nm, representing crystalline β-phase of PFO was observed in all absorption spectra of the ternary-blended thin films [25,26,27]. The constant presence of this peak proved that the existence of β-phase in the blends was unaffected. Based on both general observations, it appeared that the absorption capacity of the ternary blends is insensitive to the tiny additions of acceptors [19]. However, close observation revealed the formation of a new broad absorption peak. The new peak centered at 480 nm signifying the creation of a new ground-state level in the ternary-blended thin films as a result of F8BT additions (Figure 3 inset). A similar observation has been reported earlier [15]. 

**Figure 4:**
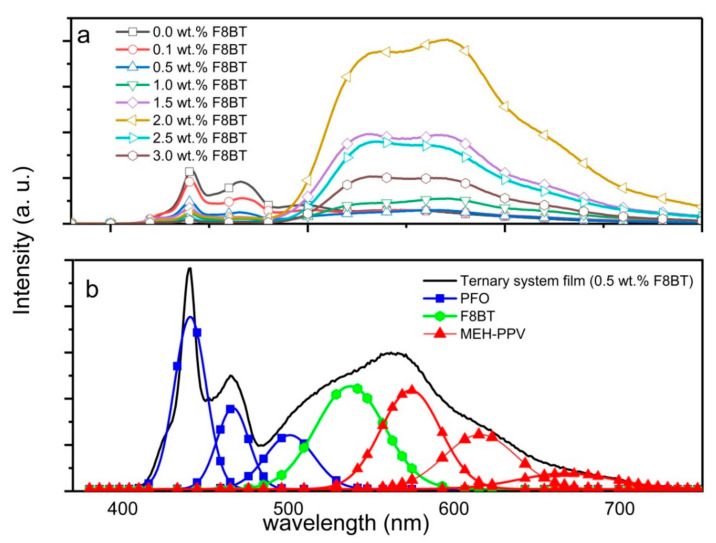
(**a**) Emission spectra of PFO/F8BT/0.3 wt.% MEH-PPV ternary blend thin films with different wt.% of F8BT; (**b**) Gaussian deconvolution of the emission spectrum from PFO/0.5 wt.% F8BT/0.3 wt.% MEH-PPV ternary blend thin film.

However, unlike the absorption spectra, more dramatic changes were recorded in the emission spectra of the ternary-blended thin films (Figure 4a). Although the additions of F8BT had resulted in a systematic reduction of the blue emission of PFO, the respective green and red emissions of F8BT and MEH-PPV behaved differently. Up to 0.5 wt.% of F8BT, there was no meaningful variation in the intensities of green and red emissions. However, at higher F8BT content (up to 2.0 wt.%), both the green and red emissions remarkably intensified. Detailed observation proved that the red emission of MEH-PPV displayed greater enhancement than the green emission from F8BT. This is because more excitons were formed in the MEH-PPV molecules since they can directly receive energy transferred from both PFO and F8BT molecules through the FRET mechanism.

An opposite emissions intensities trend was recorded once F8BT exceeded 2.0 wt.%. All emissions from the three polymers were continuously quenched thus signaling that the ternary blend system had exceeded its optimum ratio limit. Furthermore, at 3.0 wt.% of F8BT, a complete energy transfer from PFO was observed as opposed to 5.0 wt.% reported in the PFO/F8BT binary blend [28]. This is because in the ternary blend PFO/F8BT/MEH-PPV, PFO donated its energy to both acceptors. Despite complete energy transfer, the successive reductions in intensities for green and red emissions were recorded in both thin films. These reductions are likely due to the exciplexes (heterodimerics) creation at high acceptor concentration [19]. Besides, it could also be due to the active self-quenching in the blends resulting from the creation of excimers (homodimers) between acceptor monomers [29] which then converted into heat. Nonetheless, the current work successfully proves that the emission characteristics of the PFO/F8BT/MEH-PPV ternary blend can be properly tuned by carefully controlling the F8BT content.

It is imperative to note that all ternary blend thin films with F8BT contents exceeding 0.5 wt.% are not suitable as a layer for white emitters. This is because their emission spectra lack the blue emission. Instead, the desired white emission spectrum was achieved at F8BT content of 0.5 wt.% where the emissions of blue, green and red were comparable. This observation was proven by the Gaussian deconvolution of the emission spectrum from PFO/0.5wt.%F8BT/0.3wt.%MEH-PPV ternary blended thin film (Figure 4b). 

It is particularly noteworthy to mention that at F8BT contents of 0.5 wt.%, PFO has lost more than half of its initial intensity but no noticeable enhancements were recorded in the emissions of the acceptors. Although PFO continued to give away its energy to acceptors, some of the energies may be lost due to the creation of exciplexes between the ground states of F8BT monomers and the excited state of PFO monomers [15,30] and heat. The creation of exciplexes could be the primary reason for the existence of the broad peak centered at 480 nm in the absorption spectra of the ternary-blended thin films. 

#### 3.2.2. Effect of TiO_2_ Nanoparticles

One of the important data that can be extracted from Figure 4 is that the emission intensities of the acceptors can be enhanced if the donor’s emission quenching can be reversed. The reversal quenching trend in emission of donor has been observed in different binary blend thin films [8,19,20]. In these works, the general emission of the binary blend thin film was remarkably improved. This condition was possible with additions of selected metal oxides nanoparticles, in particular TiO_2_. 

In the current work, the influence of TiO_2_ nanoparticles on the photophysical properties of PFO/0.5 wt.%F8BT/0.3 wt.% MEH-PPV ternary blend thin films was systematically investigated. In addition, the dependency of current density and turn-on voltage on TiO_2_ nanoparticle contents were also examined. The nanoparticles were added incrementally to a maximum of 30 wt.% to form nanocomposite blends before being deposited onto substrates to form thin films.

**Figure 5:**
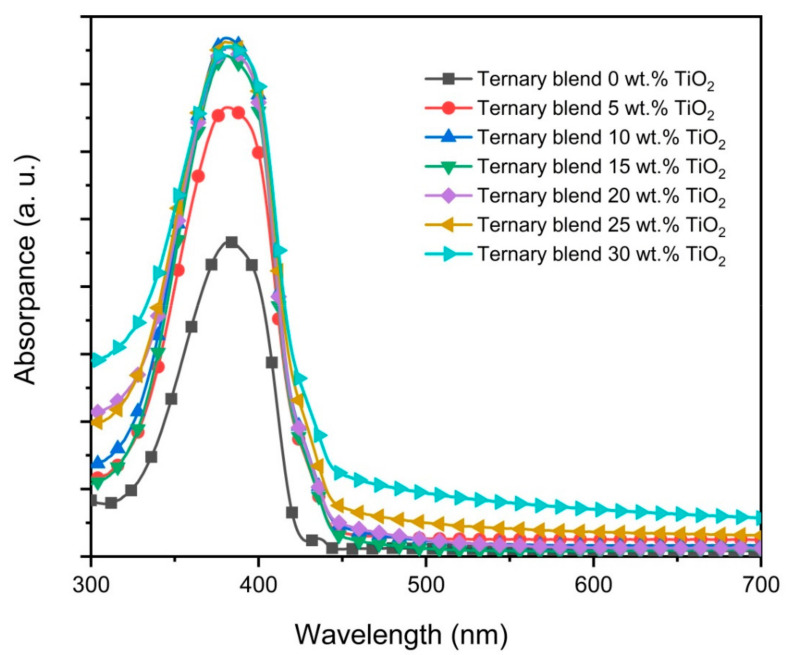
Absorbance spectra of PFO/0.5 wt.% F8BT/0.3 wt.% MEH-PPV films without and with TiO_2_ nanoparticles.

The absorption spectra of the ternary blend thin films and its nanocomposite counterparts are presented in Figure 5. There are four major observations that can be made based on these absorption spectra. Firstly, the main absorption peak at 380 nm, which refers to π–π^*^ transition in PFO, is essentially not affected by the additions of nanoparticles, signifying no alteration in its conjugation length [31,32]. Secondly, the intensity of the main absorption peak was significantly enhanced with the additions of nanoparticles up to 10 wt.%. Although further additions had a negligible effect on the absorption capacity, the initial enhancement nonetheless proved that TiO_2_ nanoparticles can be added into these blended thin films to harvest more energy from light. A similar observation was reported in an earlier work [8]. Thirdly, the small peak at 435 nm, which refers to the β-phase of PFO, disappeared with the addition of the nanoparticles. Li et al. [25] demonstrated that the crystallization of the β-phase depends on the dimensions of chain aggregation of PFO molecules. In their work, they proposed a minimum length of 100 nm. Figure 3 proves that the β-phase continues to be present in all ternary blend thin films. However, the presence of β-phase disappeared once TiO_2_ nanoparticles were added into the ternary blend signaling the breaking of the PFO molecules into shorter chains. The effect of the inorganic nanostructure on the polymer crystallinity was also proven in previous studies [33,34]. Finally, the broad peak centered at 480 nm as the signature of ternary-blended thin films had completely disappeared in all nanocomposite absorption spectra. This could be interpreted as the additions of TiO_2_ nanoparticles restricting the creation of the exciplexes between the polymers molecules and resulting in no new ground-state level. Consequently, the quenching effect of the heterodimers was inhibited as discussed below. A similar observation has been reported in an earlier work [19].

**Figure 6:**
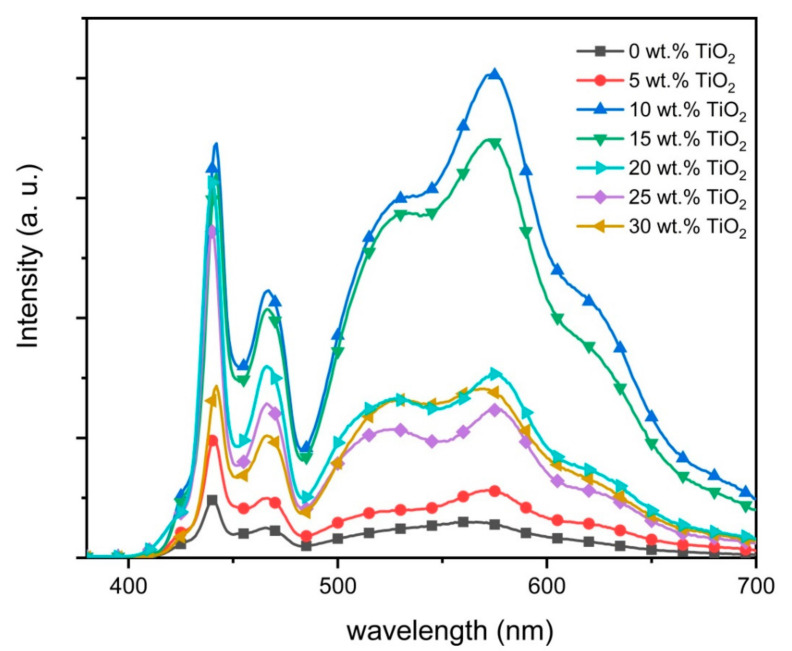
Emission spectra of PFO/0.5 wt.% F8BT/0.3 wt.% MEH-PPV/TiO_2_ nanocomposite films with different wt.% of TiO_2_ nanoparticles.

Figure 6 shows the emission spectra from all nanocomposite thin films. With additions of 5.0 wt.% TiO_2_ nanoparticles, the emissions from the three CPs in the nanocomposite film were markedly enhanced (Figure 6). Interestingly, additions of 10 wt.% of TiO_2_ nanoparticles dramatically enhanced all the emissions to the highest intensities. While the PFO emissions were essentially unchanged for TiO_2_ nanoparticles content between 10 and 20 wt.%, the reversal trend for acceptors’ emission was observed. Noticeably, the emission intensity for the PFO started to decrease at higher contents of nanoparticles. A substantial reduction can be clearly seen when the nanoparticles content in the ternary blend was 30 wt.%.

Although oxygen vacancies have been proposed as one of the fundamental reasons for the emission intensity enhancements in the nanocomposite thin films, the most widely accepted explanation is the electron trapping effect [8]. In this effect, the TiO_2_ nanoparticles, that have higher electron affinity and a lower conduction band than the LUMOs of all the polymers in the ternary blend (Figure 2), acted efficiently as a trap for the excited electrons and thus allowing more electrons in PFO to be excited. Consequently, more excitons were formed in the nanocomposite thin film which in turn results in more recombination and thus enhanced emission intensity [35]. Efficient FRET in the nanocomposite thin films was visibly manifested by improved F8BT and MEH-PPV emissions.

The decrease in the emission intensities for all CPs at high wt.% of TiO_2_ nanoparticles sent a strong signal that quenching inhibition does not depend solely on the number of nanoparticles. While the high number of nanoparticles is commendable, the effectiveness of the TiO_2_ nanoparticles to trap the excited electron also depends on the compatible energy diagram and contact quality primarily with the PFO [19,36].

**Figure 7:**
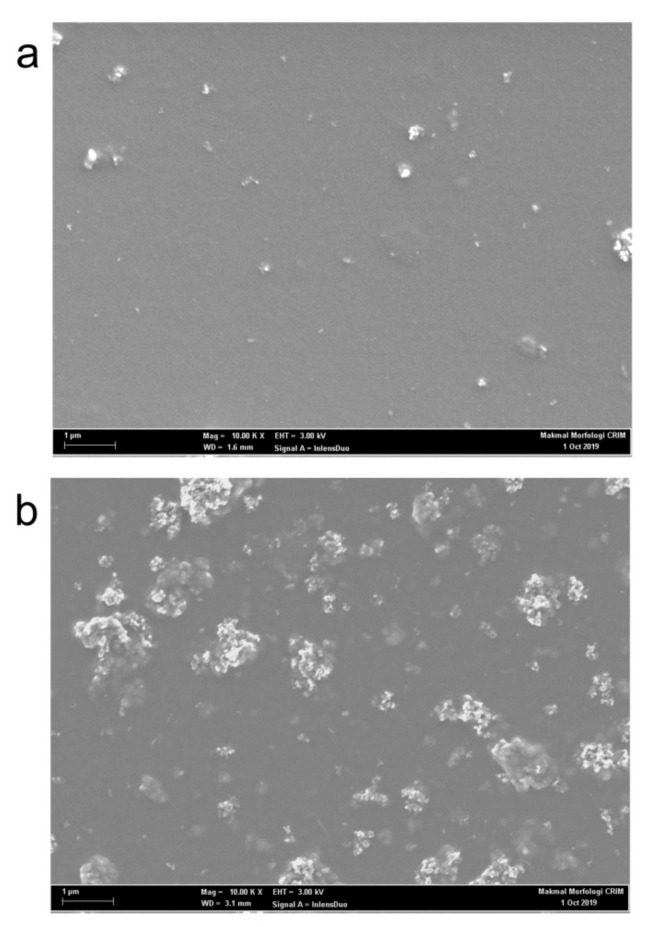
FESEM images of PFO/0.5 wt.% F8BT/0.3 wt.% MEH-PPV/TiO_2_ nanocomposite films: (**a**) 10 wt.% TiO_2_; (**b**) 30 wt.% TiO_2_.

A closer inspection of the spectra also revealed a similar enhancement trend of the blue emission shoulder centered at 423 nm. This shoulder was maximized at 10 wt.% of TiO_2_ before gradually disappearing at higher contents. Earlier work argued that this peak belongs to short chains of PFO [26]. The enhancement of this peak could indicate the presence of a significant portion of short polymer chains in PFO as result of small additions of TiO_2_ nanoparticles. This supports the idea, which was discussed above, that the presence of nanoparticles prevents the creation of long PFO chains. Furthermore, this observation can be used as a spectral proof for the nanoparticles aggregation as confirmed from FE-SEM micrographs (Figure 7) and consequently the lesser effect on PFO molecules.

Figure 7 shows the microstructural comparison between the nanocomposite thin films with the additions of 10 and 30 wt.% of TiO_2_ nanoparticles. At low content, the nanoparticles were uniformly distributed throughout the thin film creating larger contact areas with the CPs (Figure 7a). Larger contact areas enable effective electron trapping effects and thus resulted in intensified emissions. However, at the highest content, the nanoparticles exhibited strong agglomeration tendency. Large, white clusters of nanoparticles areas can be seen clearly in Figure 7b. Consequently, the areas for contact between the nanoparticles and CPs were limited. This condition reduced the effectiveness of nanoparticles as electron traps and thus explains the reason for the dramatic reduction in emission intensities at 30 wt.% addition of TiO_2_ nanoparticles.

**Figure 8:**
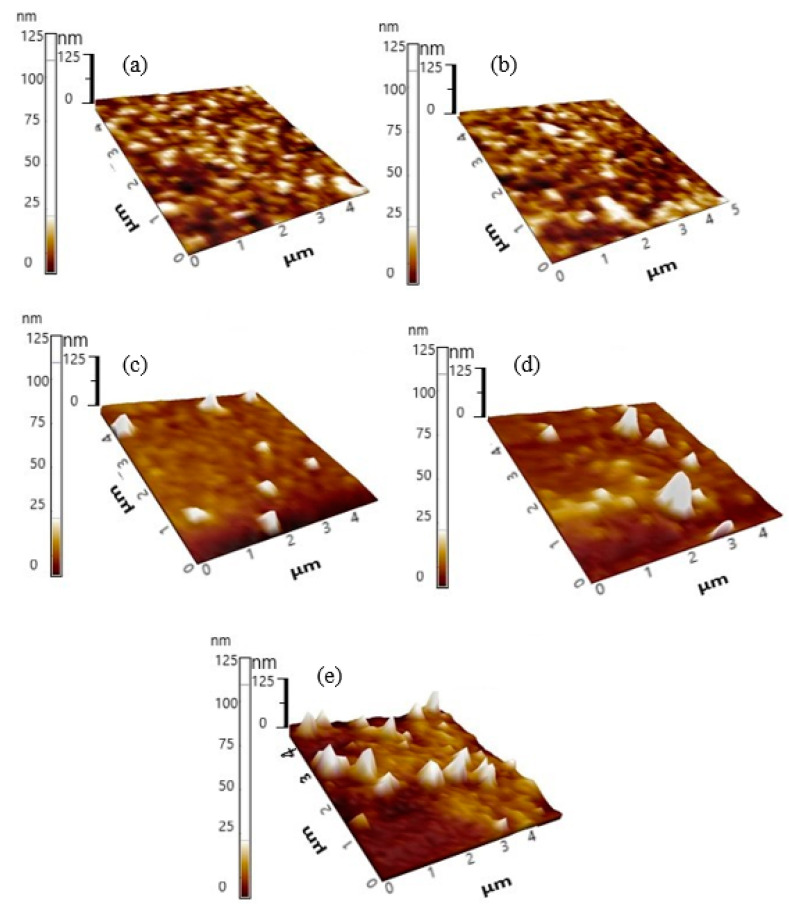
SPM images of binary, ternary and nanocomposite thin films (**a**) PFO/0.5 wt.% MEH-PPV; (**b**) PFO/0.3 wt.% F8BT/0.5 wt.% MEH-PPV; (**c**) PFO/0.3 wt.% F8BT/0.5 wt.% with 10 wt.% TiO_2_ nanoparticles; (**d**) PFO/0.3 wt.% F8BT/0.5 wt.% with 20 wt.% TiO_2_ nanoparticles; (**e**) PFO/0.3 wt.% F8BT/0.5 wt.% with 30 wt.% TiO_2_ nanoparticles.

Figure 8 shows the topography images of the binary blend, ternary blend and selected nanocomposite thin films while Table 1 summarizes the values for root mean square roughness (R_q_) and average roughness (R_a_). The binary blend thin film shows a regular smooth surface with root mean square roughness (R_q_) and average roughness (R_a_) of 1.959 nm and 1.352 nm, respectively. Apparently, the addition of 0.3 wt.% F8BT had a negligible effect on surface roughness of ternary blend thin films. Although slight increments in the values of R_a_ and R_q_ were recorded with the inclusion of 10 wt.% TiO_2_ nanoparticles, both values remained relatively low. This is because the nanoparticles were distributed uniformly throughout the film thus limiting surface defects and maintaining low phase separation. Generally, thin film surfaces with these qualities are ideal for advanced display applications such as OLEDs due to very low scattering loss, good electrode contact and enhancement of the FRET [37]. By comparison, nanocomposite thin films with higher nanoparticles contents displayed a greater tendency to agglomerate. Many large protruding white structures signifying the amplified phase separation and increase in the values of roughness were attributed to the formation of the clustered nanoparticles. While the SPM images are in good agreement with the FESEM micrographs, the rough surface topography negatively affected the FRET efficiency and thus the emissions intensities of the CPs were substantially weakened.

**Figure 9:**
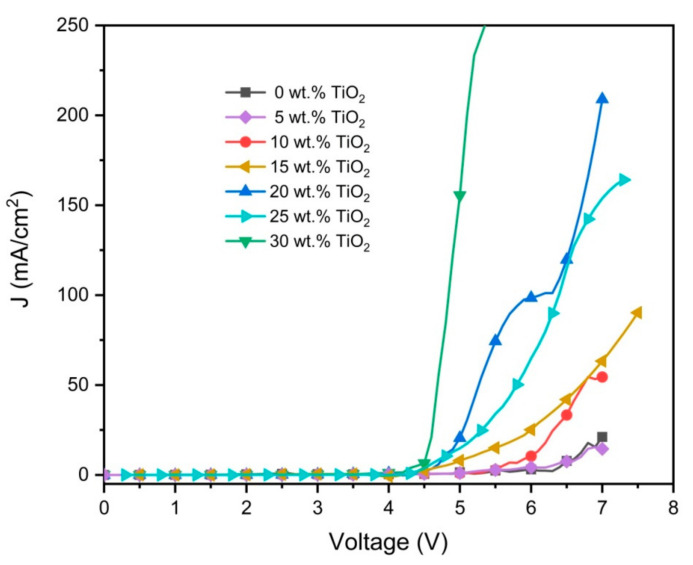
Current density–voltage (J-V) characteristics of the nanocomposite thin films.

Figure 9 illustrates the effect of the TiO_2_ nanoparticles on the J-V characteristics of the ternary blend devices. Consistent with previous studies, the additions of TiO_2_ nanoparticles increased the current density and decreased the turn-on voltage [20,38]. The increment in current density as the direct consequence of the decrement in the resistance signified the presence of a greater number of charge carriers in the nanocomposite layer [39]. Despite an increment in roughness, larger contact areas between the metal electrode (cathode) and the surface of the nanocomposite layer enable effective injection of electrons, hence lower turn-on voltages were recorded [40].

## 4. Conclusions

The effect of the acceptor ratio on the photophysical properties of the ternary blend was investigated. The photophysical properties of all ternary blends and nanocomposites thin films exhibited a strong dependency on their compositions. The desired white PL spectrum was observed when the F8BT and MEH-PPV concentrations were 0.5 wt.% and 0.3 wt.% respectively. Through the electron trapping effect, the presence of TiO_2_ nanoparticles in the ternary blend greatly intensified the emission intensities, signaling the successful inhibition of dark quenchers. Based on the current work, the optimum amount of TiO_2_ nanoparticles was 10 wt.%, contributed by uniform particles distribution and thus better contact with the CPs occurred. Severe agglomerations of nanoparticles were found to prevent further improvements although higher TiO_2_ contents had positive impacts on the J-V characteristics of the nanocomposite thin films. The PFO/F8BT/MEH-PPV blend with the optimal TiO_2_ nanoparticles displayed very encouraging properties indicating its potential for future use in the production of high-performance white OLEDs.

## Figures and Tables

**Table 1:** Root mean square roughness (R_q_) and average roughness (R_a_) values for binary, ternary and nanocomposite thin films.

Roughness	Binary	Ternary	Nanocomposite (10 wt.%)	Nanocomposite (20 wt.%)	Nanocomposite (30 wt.%)
R_q_ (nm)	1.959	2.129	2.373	8.990	10.407
R_a_ (nm)	1.352	1.424	1.612	3.184	5.106
